# Social, Structural and Behavioral Determinants of Overall Health Status in a Cohort of Homeless and Unstably Housed HIV-Infected Men

**DOI:** 10.1371/journal.pone.0035207

**Published:** 2012-04-25

**Authors:** Elise D. Riley, Torsten B. Neilands, Kelly Moore, Jennifer Cohen, David R. Bangsberg, Diane Havlir

**Affiliations:** 1 Department of Medicine, University of California San Francisco, San Francisco, California, United States of America; 2 Department of Biostatistics, University of California, Berkeley, California, United States of America; 3 Ragon Institute, Harvard University, Boston, Massachusetts, United States of America; California Pacific Medicial Center Research Institute, United States of America

## Abstract

**Background:**

Previous studies indicate multiple influences on the overall health of HIV-infected persons; however, few assess and rank longitudinal changes in social and structural barriers that are disproportionately found in impoverished populations. We empirically ranked factors that longitudinally impact the overall health status of HIV-infected homeless and unstably housed men.

**Methods and Findings:**

Between 2002 and 2008, a cohort of 288 HIV+ homeless and unstably housed men was recruited and followed over time. The population was 60% non-Caucasian and the median age was 41 years; 67% of study participants reported recent drug use and 20% reported recent homelessness. At baseline, the median CD4 cell count was 349 cells/µl and 18% of eligible persons (CD4<350) took antiretroviral therapy (ART). Marginal structural models were used to estimate the population-level effects of behavioral, social, and structural factors on overall physical and mental health status (measured by the SF-36), and targeted variable importance (tVIM) was used to empirically rank factors by their influence. After adjusting for confounding, and in order of their influence, the three factors with the strongest negative effects on physical health were unmet subsistence needs, Caucasian race, and no reported source of instrumental support. The three factors with the strongest negative effects on mental health were unmet subsistence needs, not having a close friend/confidant, and drug use. ART adherence >90% ranked 5th for its positive influence on mental health, and viral load ranked 4th for its negative influence on physical health.

**Conclusions:**

The inability to meet food, hygiene, and housing needs was the most powerful predictor of poor physical and mental health among homeless and unstably housed HIV-infected men in an urban setting. Impoverished persons will not fully benefit from progress in HIV medicine until these barriers are overcome, a situation that is likely to continue fueling the US HIV epidemic.

## Introduction

Homeless persons disproportionately suffer from serious mental and physical health problems [1] and are disproportionately infected with HIV [2]. The added burden of HIV-infection introduces further risks to overall health [3], [4] compounded by structural barriers to receiving consistent care [5], [6]. While improved antiretroviral medications have led to an era in which HIV is considered a manageable chronic condition for many individuals [7], the benefits have not been realized equally across populations due to barriers to medical care, treatment adherence and optimal health among homeless persons [2], [8], [9], [10], [11].

Few studies have examined the relative contributions of behavioral, social and structural factors influencing health outcomes over time, and even fewer have done so exclusively among community-recruited unstably housed persons. Structural factors are the policies, practices, environment and context that directly or indirectly affect an individual's options and behavior [12]. Given a variety of competing needs that are uncommon in general populations [13] and change over time, the broad economic influence of structural factors are important components of risk and risk environment among unstably housed persons. We recently reported that unmet subsistence needs had the strongest negative effect on the mental and gynecological health of HIV-infected unstably housed women, while drug use had the strongest influence on physical health (as measured by the SF-36) [14]. The aims of the current study were to determine the extent to which changing risk factors (i.e., exposure and contexts of risk) influence the physical and mental health status of HIV-infected homeless and unstably housed men over time, empirically rank risk factors by their level of influence and determine whether the most influential variables previously found among women were also the most influential variables among men.

## Methods

### Ethics Statement

All study procedures were conducted with the approval of the Committee on Human Research at the University of California, San Francisco. No funding bodies had any role in study design, data collection and analysis, decision to publish, or preparation of the manuscript.

### Sample Population

In order to include a sample of individuals who reflect San Francisco's larger population of homeless adults, methods developed by Burnam and Koegel were employed to recruit a probability sample of persons transitioning through homeless and unstably housed situations [15], [16]. From July 2002 to September 2008 a mobile outreach team recruited adults from all San Francisco homeless shelters, free food programs serving over 100 persons per day, and a random sample of single room occupancy (SRO) hotels in three neighborhoods selected with probability proportional to the number of people residing in that hotel. At small venues, all persons present on recruitment days were invited to participate in screening activities; at large venues, a subsample of individuals present (e.g., every third person) was invited to participate. Individuals who were over 18 years of age and tested HIV antibody positive were invited to enroll in the current study. Eligible individuals gave written informed consent to participate in study activities. During consent interviews, individuals were asked to answer questions regarding study procedures to address any issues of illiteracy and ensure understanding. Participants were asked to make quarterly visits (i.e., every 90 days) to a community-based field site where they provided blood for CD4 cell count and viral load assessment, and completed an interviewer-administered questionnaire to assess factors that might influence health status.

The current analysis is restricted to biological men. Restricting the population to unstably housed men allowed a focus on structural barriers that are relevant and limit options within this population. Thus, by its design, the study recognized the importance of gender inequalities and poverty as structural factors inherent to HIV and risk [17].

### Outcome Variables: Health Status

All study measures pertained to the 90 days prior to the interview. Outcome measures of self-reported health such as physical functioning and mental health are important indicators of overall health status [18]. Such self-rated health measurements offer information not consistently captured by clinical assessment [19], provide a comprehensive assessment of health status that accounts for co-occurring conditions and interactions between conditions [20], and provides significant predictors of survival [21]. The Medical Outcome Study's Short Form (SF)-36 [22] uses self-reported information to offer a reliable and valid assessment of overall health status among unstably housed individuals [23]. Two composite scores were employed in the current study, a Mental Composite Score (MCS) and a Physical Composite Score (PCS), both ranging from 0 to 100 where higher scores indicated better health.

### Main Effects: Health risks

Variables considered as having potential influence on health status included behavioral, social and structural determinants of health that have been previously established as important factors in predicting health. These factors included sociodemographic variables, including homelessness (slept on the street or in a homeless shelter); unmet subsistence needs (difficulties gaining access to housing, a bathroom, place to wash, clothing or food) [24]; social/instrumental support (having a friend who would lend the respondent money or give him a place to sleep) [25]; alcohol use (>2 drinks/day [26]); any use of heroin, crack cocaine or methamphetamine; symptoms of withdrawal from heroin, crack cocaine and alcohol, as detected in the Diagnostic Interview Schedule-IV [27]; CD4 cell count, viral load and self-reported adherence to antiretroviral therapy (ART). ART adherence was defined as 0% among persons who were ART eligible (i.e., CD4 cell count ≤350 by clinical guidelines in place during the study period [28]), but not currently taking ART. Health services were not adjusted in the current study, as service use may be a consequence of poor health and not a risk factor.

### Statistical Methods

Marginal structural models and targeted variable importance (tVIM) have been used previously to rank influences of social and structural factors on the overall health status of HIV-infected impoverished persons [14], and were employed in the current study. This approach is uniquely suited to handle time-dependent confounding of variables that are more commonly found in impoverished populations (e.g., drug use may lead to poor health, but poor health may lead to subsequent self-medicating drug use [29]).

tVIM assesses the effects of a large number of variables with unknown or diverse correlation structures; it more accurately assesses effects when compared to techniques that rely on parametric regression models [30], [31], [32], [33]. tVIM estimates the effect of one variable at a time, which tailors the estimation approach towards the specific effect of interest, thereby providing a more accurate effect and assessment of uncertainty. This approach is important for analyses described herein because different data types and a broad spectrum of variables were analyzed, thus a single multivariable regression model approach was untenable. tVIM involves two steps: First, to ensure that the exposure preceded the outcome (an assumption of risk and the statistical models that estimate it) and then fit a marginal structural model to estimate the target parameter. The marginal structural model estimated the population-level effect [34] of each risk factor in the previous quarter on health status of the current quarter (i.e., a 1 unit change for MCS or PCS), adjusting for potential confounding [35]. Variables were considered as potential confounders in all models for which they were not being considered as a primary effect, and confounders were assessed separately for each model estimating a primary effect.

Second, tVIM techniques were applied such that the risk factor-specific effects were ranked by p-value, which was appropriate for the current study due to the fact that exposure variables (i.e., risk factors) had different units of measure. Because the population and sample size were consistent between models, ranking variables based on p-value was a standardized approach to ranking effect estimates (i.e., signal to noise ratio). Thus, ranking is not from the most negative to the most positive effect, it is from the variable with the largest population-level effect on the outcome to that with the smallest.

## Results

A 3% refusal rate for study enrollment resulted in a cohort of 288 HIV-infected men. The median age of study participants at baseline was 41 years (IQR = 35–46). Less than 40% of study participants had graduated from high school, almost 60% were of non-Caucasian race/ethnicity and 23% reported the recent use of crack cocaine (Table 1). Regarding structural factors reported at baseline, 20% of study participants had slept on the street or in a homeless shelter, 8% had been incarcerated and 26% had unmet subsistence needs during the 90 days prior to baseline (Table 1). Considering a 2% annual loss to follow up and 0.5% annual mortality rate, the median follow-up time was 15 months per person.

**Table 1:** Baseline Characteristics of HIV-Infected Homeless and Marginally Housed Men living in San Francisco, CA, USA, 2002–2008 (N = 288).

SOCIOECONOMIC	
Age	Median = 41 (IQR = 35–46)
Graduated from high school	38%
Race/ethnicity	
African American	38.4%
Caucasian	41.2%
Latino	7.4%
Other	13%
Any minor children living at home (past 90 days)	0.36%
Incarcerated (past 90 days)	8%
Slept on the street or in a homeless shelter (past 90 days)	20%
**SUBSISTENCE NEEDS AND SOCIAL SUPPORT (past 90 days)**	
Employed	7%
Any income from SSI, SSDI	65%
Current monthly income	Median = $815 (IQR = 690–878)
Unmet subsistence needs <$>\raster="rg1"<$>	26%
Has close friend/confidant	68%
No reported instrumental support	28%
**DRUG AND ALCOHOL USE (past 90 days)**	
Crack cocaine	23%
Heroin	12%
Methamphetamine	2%
Alcohol use (>2 drink/day)	9%
MENTAL HEALTH	
CD4 cell count	Median = 349 cells/µl (IQR = 178–546)
Viral load	Median = 7,200 copies/ml (IQR = 15–43,250)
Current symptoms of any chronic health condition ‡	33%
Overall Physical Health score (PCS)◊	Median = 43 (IQR = 33–50)
**MENTAL HEALTH**	
History of depression	35%
History of schizophrenia	3%
History of manic episodes	22%
History of PTSD	16%
Overall Mental Health Score (MCS)◊	Median = 46 (IQR = 33–55)

<$>\vskip-2\scale60%\raster="rg1"<$>Access to a bathroom, place to wash, clothing, food and a safe place to sleep.

‡ Asthma, diabetes, heart disease, high blood pressure or emphysema.

◊ Out of 100 where a higher score indicates better health.

### Physical Health

At baseline, the median viral load in this sample population was 7200 copies/ml, the median CD4 cell count was 349 cells/µl and 18% of eligible persons (CD4<350) took antiretroviral therapy. Over one-third of study participants reported current symptoms of chronic illness (Table 1). The population median for overall physical health (PCS) was 43 (out of 100 possible). Adjusting for all significant study confounders, unmet subsistence needs was the most important explanatory variable (i.e., had the largest effect) among the study's estimated effects on the overall physical health of men in this sample (Table 2). On average, men reporting unmet subsistence needs had PCS scores that were 3.8% lower (p = 3.4e-05) than those who did not, after adjusting for all other significant study confounders. In separate models, and in order of their adjusted population effect on physical health status scores, Caucasian participants had PCS scores that were 3.7% lower (p = 1.2e-03) than other racial/ethnic categories; those with no instrumental support had scores that were 1.6% lower (p = 2.2e-02), and for every unit increase in viral load, PCS scores decreased −1.8e-05% (p = 4.1e-02).

**Table 2:** Ranked Influence of a Unit Increase in Competing Needs during the past 90 Days on the Overall **Physical Health** (Score 0–100)* of HIV-Positive Homeless and Unstably housed Men, 2002–2008 (N = 288)‡.

Main Effect	Crude Population Effect	Crude 95% Confidence Interval	Crude p-value	Adjusted Population Effect	Adjusted 95% Confidence Interval	Adjusted p-value	tVIM Rank
Unmet subsistence needs	−4.33	(−6.06,−2.59)	1.4e-06	−3.83	(−5.27,−1.6)	3.4e-05	1
Caucasian race/ethnicity	−3.71	(−6.08,−1.31)	2.2e-03	−3.71	(−6.03,−1.29)	1.2e-03	2
No source of instrumental support	−1.74	(−2.94,−0.56)	4.2e-03	−1.56	(−2.88,−0.21)	2.2e-02	3
Viral load	−1.8e-05	(−3.8e-05,−3.1e-06)	4.5e-02	−1.8e-05	(−3.8e-05,−3.1e-06)	4.1e-02	4

* SF-36 Physical Health Composite Score (PCS) in which a higher score indicates better health.

‡ Each line represents a separate model in which a single main linear effect is estimated, adjusting for the potential confounding of all other significant study variables.

### Mental Health

Regarding individual mental health conditions, 35% of the population had a history of major depression, 22% experienced manic episodes and 16% had a history of post-traumatic stress disorder (PTSD) (Table 1). The population median for overall mental health (MCS) was 46 (out of 100 possible). Adjusting for all significant study confounders, unmet subsistence needs was the most important explanatory variable among the study's estimated effects on the overall mental health of men in this sample (Table 3). On average, men reporting unmet subsistence needs had MCS scores that were 3.5% lower than those who did not (p = 3.6e-05), after adjusting for all other significant study confounders. In separate models, and in order of their adjusted population effect on MCS, individuals reporting a close friend/confidant had MCS scores that were 3.2% higher (p = 4.5e-05), while MCS scores were 3.7% lower (p = 2.0e-04) among drug users, 2.2% lower (p = 1.2e-03) for those with no instrumental support and 1.7% higher (p = 4.3e-02) for those with ≥90% ART adherence.

**Table 3:** Ranked Influence of a Unit Increase in Competing Needs during the past 90 Days on the Overall **Mental Health** (Score 0–100)* of HIV-Positive Homeless and Unstably housed Men, 2002–2008 (N = 288)‡.

Main Effect	Crude Population Effect	Crude 95% Confidence Interval	Crude p-value	Adjusted Population Effect	Adjusted 95% Confidence Interval	Adjusted p-value	tVIM Rank
Unmet subsistence needs	−5.35	(−7.25,−3.42)	4.9e-08	−3.51	(−5.08,−1.29)	3.6e-05	1
Has a close friend/confidant	2.36	(0.91,3.82)	1.6e-03	3.19	(1.64,4.72)	4.5e-05	2
Any drug use	−4.45	(−6.41,−2.45)	1.2e-05	−3.67	(−5.53,−1.8)	2.0e-04	3
No reported sources of instrumental support	−1.81	(−3.11,−0.53)	7.0e-03	−2.2	(−3.62,−0.89)	1.2e-03	4
≥90% ART adherence	3.32	(1.37,5.18)	7.3e-04	1.66	(0.07,3.27)	4.3e-02	5

* SF-36 Mental Health Composite Score (MCS) in which a higher score indicates better health.

‡ Each line represents a separate model in which a single main linear effect is estimated, adjusting for the potential confounding of all other significant study variables.

After adjusting for all significant confounders, variables that were not among the strongest predictors of overall mental and physical health included age, race, income and CD4 cell count.

## Discussion

Among HIV-infected homeless and unstably housed men who were aware of their HIV status and eligible for treatment in a resource-rich environment, only 18% took ART at baseline. Moreover, while ART adherence and viral load were among the most important predictors of overall health, unmet subsistence needs and social support had even larger influences in this population. These results are based on six years of follow-up, during which time detailed longitudinal data were obtained on a probability sample of 288 individuals, making it one of the most thorough and extensive data sets of its kind. Every exposure examined was established in previous research as important to the health of unstably housed individuals. With overwhelming burdens of illness experienced by homeless persons and limited resources to address these issues, health care and social service providers are often left with the responsibility of choosing which important factor to prioritize. Results presented here suggest that addressing basic subsistence needs first (i.e., ensuring access to housing, food, clothing and hygiene needs) will have the most impact on the health of HIV-positive unstably housed persons. Thus, advances in medical science that are saving, lengthening and improving the quality of life for many people living with HIV/AIDS will not fully benefit unstably housed persons until their basic subsistence needs are met.

Results presented here expand implications from a recent CDC report showing that poverty is the single most important demographic factor associated with HIV infection among inner-city heterosexuals living in the United States [36]. Taken together, these observations indicate that unmet subsistence needs are having critical influences on the health of impoverished persons both infected with and at risk for HIV/AIDS, which is consistent with findings from multiple HIV outcomes studies. For instance, homelessness is a risk factor for both HIV acquisition [37] and delayed diagnosis among men who have sex with men [38], a strong predictor of initiating injection drug use [39], [40] as well as unsafe syringe acquisition and disposal [41], a significant correlate of transactional sex [42], [43] and unprotected sex among high-risk heterosexual women [44]. It is clear that the influences of poverty on the US HIV epidemic are not confined to exceptional cases, nor are they confined to sub-groups. Poverty is a pervasive force driving the epidemic and its influences on health.

How to address poverty as a leading cause of morbidity is a source for ongoing debate worldwide, including resource-rich countries like the United States [45]. While research is rarely able to measure moral dimensions of homelessness such as dehumanization, diminished capacity to actualize basic societal rights and privileges, and susceptibility to victimization [46], a variety of studies have shown measureable health improvements from structural interventions. Specifically, studies evaluating the effects of housing and case management have demonstrated significant reductions in medical care utilization and improvements in physical and mental health [47], [48], [49]. Such interventions have also been shown to offset costs of acute care and significantly decrease overall costs [46], [50], [51], [52]. In short, while regional variations exist, homelessness is more expensive to society than the costs of permanent housing [46]. Similarly, research has shown that the Supplemental Nutrition Assistance Program (SNAP) decreases food insecurity by 20–50% [53], and the Expanded Food and Nutrition Education Program (EFNEP) translates into a positive cost-benefit based on potential prevention of diet-related chronic diseases and conditions [54]. Considered in association with results presented here, these studies suggest that subsistence needs such as housing and food insecurity have the most influence on the overall health of HIV-positive unstably housed persons and can be successfully intervened upon. Taken together, this body of empirical evidence suggests that social programs addressing subsistence needs are fiscally sound.

The low level of ART use and strong influence of ART adherence on health in the current study are particularly relevant in light of recent dialogues regarding expanded HIV treatment. Theoretical decreases in HIV incidence from expanded treatment [55] have been interpreted with caution in the social context of the US HIV epidemic [56] on the grounds that ART availability and use are determined by a multi-faceted and interrelated array of clinical, epidemiological, biological, social and behavioral factors. In this context, the use of ART may be lower than expected and thus theoretical reductions in HIV incidence from expanded treatment may be limited in certain populations such as those experiencing extreme poverty. Findings presented here support and extend this position as follows: the use of ART is a multi-faceted phenomenon; the overall health of HIV-infected impoverished persons is also a multifaceted phenomenon and relies neither exclusively nor primarily on ART.

Strong connections exist between poverty, structural factors, poor health and non-Caucasian race/ethnicity in the United States. The finding that Caucasian race/ethnicity predicted worse health was thus unexpected and contradicts medical research conducted in the general US population [57], [58] as well as the general US HIV/AIDS population [59]. However, contrary to the general US HIV epidemic, the recent CDC analysis found no significant differences in HIV prevalence by race/ethnicity when data were considered from exclusively low-income areas [36]. Data reported here do not only apply to low-income individuals, but individuals who live in such extreme poverty as to be without stable housing. These results thus extend CDC findings and suggest that, when data are restricted to extremely impoverished persons, effects of race/ethnicity may not only be diminished relative to the general US HIV epidemic, but there may be situations in which effects are in the opposite direction. The mechanism by which HIV-infected unstably housed men of color experience better overall health compared to Caucasian HIV-infected unstably housed men cannot be established with these data and warrants additional inquiry. In particular, future studies that assess associations between race and length of time living with HIV, and the mediation of these influences by health services use, would facilitate a better understanding of this effect.

Comparing results from the current analysis to our previous work regarding the health status of HIV-infected unstably housed women, there are two main points of divergence. First, race/ethnicity was not among the most influential predictors of health status among women [60]. Second, after adjusting for basic subsistence needs, street homelessness was among the strongest predictors of worse overall health among women [60], while this effect was not as strong for men in the current study. On the other hand, the most influential variable in both gender-specific cohorts is basic subsistence needs. The consistency and strength of this finding provides evidence that prioritizing basic subsistence needs (i.e., housing, food, clothing and the use of a bathroom) would lead to the largest population-level health improvements among extremely impoverished HIV-infected persons living in the US.

The results of this study should be considered in light of potential limitations. First, study participants may have underreported behaviors such as drug use, due to social desirability; however, this would have biased results toward the null, indicating that effect sizes are at least as extreme as those reported. Second, data were taken from a single well-resourced metropolitan area and generalizability may be limited. There is, however, evidence suggesting similar findings regarding influences of poverty and housing on health in other metropolitan areas [11], [61], [62], [63], [64], thus, influences of location are likely minimal. Third, models used in this study assumed that there were no unmeasured confounders related to health status, and it is possible that residual confounding existed from unmeasured effects. This limitation is inherent to all traditional modeling techniques and our inclusion of factors that have been found by previous studies to be important correlates of health status was intended to minimize this potential limitation. Fourth, results suggesting that ART adherence positively influences mental health may not represent the true causal pathway (e.g., baseline mental health influences adherence and not the other way around); however, a marginal structural model approach was chosen specifically to address these complicated associations. With IPTW estimation, weights create a pseudo-population in which the previous mental health outcomes are no longer confounders, which allows the construction of an unbiased estimator for the parameter of interest. Results presented here therefore indicate that, after accounting for influences of mental health on ART adherence, individuals with high levels of adherence had overall mental health scores that were an average of 3% higher.

Results presented here and in our earlier women's study [60] indicate that unmet subsistence needs have the largest population-level effects on the mental and physical health of unstably housed HIV-positive individuals and that the biggest population-wide impact on health would be made by focusing on these issues. Given that the influences of poverty and housing instability on the US HIV epidemic are pervasive throughout major risk groups [37], [38], [39], [40], [41], [42], [43], [44], addressing subsistence needs stands to have broad impact on overall health. Furthermore, given the US Census Bureau's recent report indicating that the nation's poverty rate rose more than 15% last year, resulting in 46 million impoverished people living in the United States [65], this impact is likely growing.

While a combination of behavioral, biomedical and structural interventions is expected to provide the most effective approach to HIV prevention [66], [67] and HIV treatment, advances in HIV medicine will not be fully realized by unstably housed persons until opportunity and choice limited by social and structural barriers are overcome. Moreover, the social and structural barriers inherent in poverty are not only likely to continue fueling the US HIV epidemic until they are overcome, but they now have opportunity to do so at a faster rate with currently increasing rates of US poverty.
